# Survivability Is More Fundamental Than Evolvability

**DOI:** 10.1371/journal.pone.0038025

**Published:** 2012-06-18

**Authors:** Michael E. Palmer, Marcus W. Feldman

**Affiliations:** Department of Biology, Stanford University, Stanford, California, United States of America; Queen Mary, University of London, United Kingdom

## Abstract

For a lineage to survive over long time periods, it must sometimes change. This has given rise to the term *evolvability*, meaning *the tendency to produce adaptive variation*. One lineage may be superior to another in terms of its current standing variation, or it may tend to produce more adaptive variation. However, evolutionary outcomes depend on more than standing variation and produced adaptive variation: deleterious variation also matters. Evolvability, as most commonly interpreted, is not predictive of evolutionary outcomes. Here, we define a predictive measure of the evolutionary success of a lineage that we call the *k*-survivability, defined as the probability that the lineage avoids extinction for *k* generations. We estimate the *k*-survivability using multiple experimental replicates. Because we measure evolutionary outcomes, the initial standing variation, the full spectrum of generated variation, and the heritability of that variation are all incorporated. Survivability also accounts for the decreased joint likelihood of extinction of sub-lineages when they 1) disperse in space, or 2) diversify in lifestyle. We illustrate measurement of survivability with *in silico* models, and suggest that it may also be measured *in vivo* using multiple longitudinal replicates. The *k*-survivability is a metric that enables the quantitative study of, for example, the evolution of 1) mutation rates, 2) dispersal mechanisms, 3) the genotype-phenotype map, and 4) sexual reproduction, in temporally and spatially fluctuating environments. Although these disparate phenomena evolve by well-understood microevolutionary rules, they are also subject to the macroevolutionary constraint of long-term survivability.

## Introduction

*O species, stunned by your terror of chill death, why fear the Styx, why fear the ghosts and empty names, the stuff of poets, the spectres of a phantom world? […] Everything changes, nothing dies: the spirit wanders, arriving here or there, and occupying whatever body it pleases, passing from a wild beast into a human being, from our body into a beast, but is never destroyed. As pliable wax, stamped with new designs, is no longer what it was; does not keep the same form; but is still one and the same.*

- Ovid, Metamorphoses, book XV: 143–175

### Evolvability

“Evolvability” has been much discussed in the last 20 years, but there is no agreement over what this term should mean [1]. Kirschner and Gerhart [2] call evolvability, “an organism’s capacity to generate heritable phenotypic variation,” without reference to the fitness of that variation. Houle [3] associates evolvability with current standing variation in the population (described by the “G-matrix”), which relates it to the immediate response to selection of a population. In contrast, Wagner and Altenberg [4] propose that the qualitative meaning of evolvability should reflect the “the genome’s ability to produce adaptive variants when acted upon by the genetic system.” In this sense, evolvability is about the adaptive variation that *may be produced*, rather than the *current* standing variation. Jones et al. [5] note that the capacity to produce variation is related to the “M-matrix” used in quantitative genetics; however, like Wagner and Altenberg, they restrict evolvability to the production of adaptive variation only, and offer no quantitative definition of evolvability.

The above authors refer to many different entities as possessing evolvability: Kirschner and Gerhart [2] see it as a property of organisms. Houle [3] and Jones et al. [5] see it as a property of populations. Draghi and Wagner [6], [7] and Palmer and Feldman [8] see genotypes as the entities that possess evolvability. Wagner and Altenberg [4] in one place call evolvability a property of “the genome” and in another they suggest that it is a property of the evolutionary “system”.

Attempts to define evolvability quantitatively are few, and have been applicable only to a particular model. For example, Draghi and Wagner [6] measure evolvability as the mean decrease in “Hamming distance” between a binary vector phenotype and all 16 possible target vectors in their model, within a time span of 25 generations. Similarly, Griswold [9] defines evolvability as the “rate of evolution of a phenotypic character,” in units specific to the phenotypic character, and invokes a specific time span of 10,000 generations. In another model with a genotype comprising two genetic vectors, evolvability is measured as the tendency for these vectors to be oriented perpendicular to one another [7], [8] after a fixed number of generations.

None of the above definitions of evolvability can be used to predict evolutionary outcomes. If only standing variation is considered, then new variation, which is clearly important to long-term outcomes, is ignored. If only new variation is considered, then the importance of standing variation in the short term is neglected. A lineage may have excellent potential to *produce* adaptive variation, given sufficient time, but may go extinct in the short term due to inappropriate initial standing variation. While the production of *adaptive* variation may be important, the production of *deleterious* variation should also be taken into account. If any of these factors is left out, it may be impossible to predict evolutionary outcomes.

### Predictive Metrics of Short-term and Long-term Evolutionary Success

We suggest that the underlying goal of research on evolvability has been to distinguish predicted evolutionary success in the *short term* from that in the *long-term*. Rather than attempting to quantify evolvability using the above definitions, we define two quantitative metrics that do predict evolutionary outcomes, and do permit us to distinguish short- and long-term predicted evolutionary success. The first of these metrics, called the *k-fitness*, measures the predicted “success” of a lineage as the expected factor of increase in number of members, over *k* generations; this the standard absolute fitness of a lineage extended to multiple generations. The second metric, called the *k-survivability,* measures the predicted “success” of a lineage as the likelihood that it will survive (avoid extinction) for *k* generations. Because these metrics summarize a set of realized evolutionary outcomes, they include the effects of: 1) initial standing variation, 2) produced variation, 3) adaptive, deleterious, and neutral variation.

The standard, one-generation, absolute fitness is defined as the expected factor of increase in the number of copies of (for example) a genotype, over a single generation. The fitness of a genotype can be estimated using multiple replicate experiments, under “similar conditions” (defined precisely below) in each replicate. Importantly, the fitness is (sometimes) predictive. We will call the fitness *predictive* if, in a new replicate of the experiment, we have a certain confidence that the factor of increase will be near the previously estimated mean. Otherwise, we cannot be confident that the factor of increase in another replicate experiment will be near the previously measured mean. For many interesting traits and situations, the standard one-generation fitness is predictive.

The *k*-fitness, the expected factor of increase in the membership of a lineage over *k* generations, can also be estimated in multiple replicate experiments. Like the one-generation fitness, the *k*-fitness may sometimes be predictive. If so, then a new replicate of the experiment in a sufficiently similar evolutionary context (defined precisely, below) is again expected to increase in membership by a similar factor. Our second metric, the *k*-survivability, the probability that a lineage will survive for *k* generations, may also be predictive, depending on the context. Below, we describe an entropy-based metric of predictability.

### Lineages

Following Hull [10], we focus on the *lineage* as the entity that evolves. A lineage may persist indefinitely (until it goes extinct). The individual members of a lineage at one generation give rise to its individual members at the next generation. A lineage may accumulate genetic change, yet still retain its identity through the relationship of descent. We define a lineage as a founding population of organisms (or a single founding individual, in asexuals) and zero or more generations of its descendant populations (or descendant individuals, in asexuals). Thus a lineage is monophyletic.

For sexuals, we assume that *a lineage begins at an irreversible speciation event*. The founders of a sexual lineage are all the members of one of the nascent species. This definition implies that *two distinct lineages cannot fuse together*. A lineage can continue to speciate, however, and all members of sub-lineages thus formed remain members of the original lineage. (This assumption clarifies the assignment of “credit” for numerical increase, or for survival: if we permitted two lineages A and B to fuse, then a vanishing genetic contribution from lineage A would allow it to “hitchhike” on the success of lineage B.) Thus a sexual lineage at its foundation consists of exactly one population, but may later include multiple distinct populations and/or species. The founding requirement is automatically satisfied for asexuals: every asexual individual is the founder of a lineage (as well as a member of each lineage to which its parent belonged).

### Persistence of the Lineage is Fundamental; Number of Members is Not

Although increase in the number of individual members of a lineage may lower its chance of extinction, this is only one of several possible ways to enhance survivability. The genetic and spatial distributions of the individual members of a lineage are both important to its survivability. Random mutation sometimes leads to a phenotype that is well suited to the environment. Similarly, random *migration*, or dispersal, may result in an organism moving to an environment where its phenotype has a higher probability of survival. When a lineage disperses in space, or diversifies in lifestyle, the likelihood that all its members will die may be decreased, tending to increase the survivability of the lineage. The more dispersed and diversified the lineage, the less it will matter that some of its members have low fitness at some places and times.

Gould and Lloyd [11] have insisted that entities that experience selection “must generate offspring (in potentially differential numbers…),” but we disagree on this technical detail. Two lineages have experienced relative selection if the “winner” survives, and the “loser” goes extinct. It is not important whether either lineage produces many sub-lineages, or many individual members, but it is fundamental that the extinction of one, and the survival of the other, are realized. Long-term “successful” lineages are not necessarily those that speciated into many distinct sub-lineages, but *those that avoided extinction*.

### Levels of Selection; Must there be Only One?

The “Lloyd-Vrba debate” [12] concerned what phenomena should qualify as selection at the species level (in this article, the lineage level), as distinguished from selection at the individual level. Lloyd [13] requires that species fitness not be reducible to the effects of individual fitnesses; Vrba’s [14] definition is even more restrictive. Since we are primarily concerned with the survival probability of lineages, and not with distinguishing between selection at the individual, versus lineage, levels, we use “lineage selection” even if the fitness effect is reducible to effects on individuals. It is not necessary to attribute such selection *exclusively* to the individual level [15]; it may simultaneously be considered as aggregate selection on the lineage. For example, when the ornithischians went extinct, the deaths of the individual members were not statistically independent events. As a lineage, they shared aggregate risk, and suffered a shared catastrophe. Thus when we refer to “selection on a lineage” we will include both what Lloyd [13] would call strict, irreducible “species selection” and what Grantham [12], calls “effect macroevolution”, namely, the aggregate effect on a lineage of selection on its individuals.

### Selection at Different Timescales

Most previous authors have considered selection on individuals, and selection on lineages (or species), as distinct “levels” of selection. We feel it is more appropriate to consider selection on lineages, and vary the number of generations, *k*, that are considered. In the immediate term (*k* = 1), only the standing phenotypic variation in the lineage is selected. In the short term (*k*>1), the standing variation in the lineage may still dominate; however, a lineage may also produce new variation at each generation, and this is may also be subject to selection. In the long term (k≫1), the lineage phenotype may change dramatically; thus the variation that arises in a lineage during the evolutionary process becomes increasingly important, relative to the initial standing variation. Clearly, however, immediate-term selection can never be entirely ignored: a lineage must survive in the immediate term if it is to survive in the long term.

Thus, what some authors would prefer to think of as selection at the “individual” level, we would describe as selection acting on the lineage in the immediate term (*k* = 1). Even if the lineage consists of multiple individuals, or multiple populations, at a particular moment in time, we still consider the aggregate immediate selection on these larger collections to be immediate-term selection on the lineage. Framing immediate-term selection in this way permits a natural extension to longer time scales.

### Migration Versus Mutation: Two Strategies for Matching the Phenotype to the Environment

Random mutation may produce a phenotype that is well suited to the environment. Similarly, random migration, or dispersal, may move the organism to an environment that suits its phenotype. Dispersal is similar to random mutation in that too high a rate of dispersal (or mutation) to unfavorable geographic (or genetic) “neighborhoods” can reduce the chance that the lineage will survive. However, dispersal may enhance the survivability of the lineage if: 1) it moves individuals to where they are more fit, or 2) it decreases the joint likelihood of extinction among related individuals and sub-lineages. Even if each local population has a similar risk of extinction per unit time, these local extinctions are likely to be less correlated if they are geographically separated, making it less likely that all members of the lineage will die.

## Results

### Definitions: The *k*-generation Fitness and Survivability of Lineages

We define the *k-generation fitness* and *k-generation survivability* of lineages as follows:


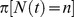
 is defined as the probability that a lineage has *n* members at generation *t*.


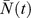
 is the expected number of members of the lineage at generation *t*, namely, 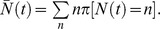



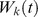
 is the “*k*-fitness” at generation *t*, namely, the ratio of the expected number of members of the lineage at generation *t*+*k*, to the expected number at generation *t.* (*t* ≥0, k ≥1.) 
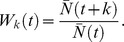



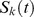
 is the “*k*-survivability” at generation *t*, namely, the probability that the lineage will survive to generation *t+k*, given that it has survived to generation *t*

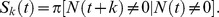



(To refer explicitly to the *k*-fitness or *k*-survivability of a lineage *i* among several lineages, we will add a superscript, as in 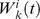, 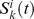, but we will usually omit it for brevity.)

Because selection on lineages is ultimately determined by extinction, the *k*-survivability, 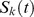, is the more fundamental measure of evolutionary success. Nonetheless, 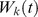 is considered here because, for short time spans, extinctions of lineages may be rare, providing little statistical power to measure the *k*-survivability. In this case, the *k*-fitness is a practical proxy for the *k*-survivability.


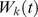
 and 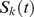 will be estimated in multiple experimental replicates, each of which begins at generation 0, and continues to generation *t*+*k*. These replicates have distribution I of initial states of the lineages at generation 0 (one “initial state” specifies the initial genotypes and the spatial distribution of the individual members of the lineages). E represents the distribution of environmental conditions experienced between generation 0 and generation *t*+*k*. Thus 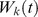 and 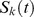 each measures the expected “evolutionary success” of a lineage by comparing the distribution of the number of members at time *t* with the distribution at time *t*+*k*, under the distributions I and E.

I and E define the evolutionary context in which we determine whether the metrics are predictive. For example, with all else being equal, 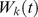
and 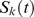 might be predictive if the initial number of members (described by the distribution I) varied within a narrow range, but not if that range were wide. Indeed, in one of our examples below, the outcomes do depend on population size. Thus we might find predictability for some distributions I and E, but not for others.

As a measure of predictability, we propose 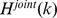, the entropy of the “joint survival state”, computed as follows. Call *s(i, j, k)* the survival state of lineage *i* in replicate experiment *j* at time step *k*. *s(i, j, k)*  = 1 if lineage *i* has any living members at time step *k* of replicate *j*, and 0 otherwise. (Thus the *k*-survivability for lineage *i*, 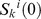, is simply the average of *s(i, j, k)* over all replicates *j*.) The “joint survival state” *M(j, k)* is a binary vector composed of elements *s(i, j, k)*, ordered by *i*. If there are *L* lineages, there are 2*^L^* possible values of the vector *M(j, k)*. By averaging over the replicates, we can estimate *p_m_(k)*, the probability that each particular joint survival state *m* will occur at time step *k*. We then compute the entropy of joint survival state:


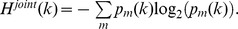
 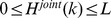 (in units of bits).

The entropy is also called the “surprisal”. 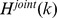 measures, on average, how surprised we will be to see the joint outcome (one of the 2*^L^* possible states) of a replicate. If 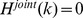, then the same state always occurs at time step *k*: the outcome is perfectly predictable; we are never surprised.

We also compute 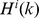, the entropy of the survival state of each individual lineage *i*. Below, we show that the individual entropies, 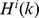, are useful for interpreting the variation in the joint entropy, 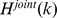, over time. 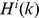 turns out to be a simple function of 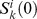, the *k*-survivability of lineage *i*:
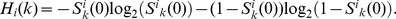



Because there are only two possible states of an individual lineage, 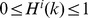. The joint entropy and the individual entropies obey the following inequalities:


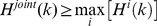
, and 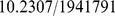


We refer to *k* = 1 as the *immediate term*. For example,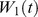 (which is the standard fitness used in population genetics) is the *immediate fitness* at generation *t*, and 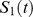 is the *immediate survivability* at generation *t*. We will refer to *k* slightly greater than one (k>1) as the *short term*, and *k*≫1 as the *long term*. For the immediate term (*k* = 1), the evolutionary outcome depends only on the standing variation in the lineage at generation *t*. As *k* increases, the spectrum of variation produced by each lineage becomes increasingly important to the outcome.

Here, we illustrate the measurement and use of 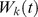 and 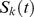 with *in silico* population genetic models. However, in principle, they may also be measured for *in vitro* and *in vivo* systems. An important requirement is that a sufficient number of replicate experiments must be conducted to estimate 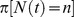, over particular distributions of both I and E, in which case we can estimate 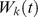 and 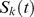. In practice, this may be possible with bacterial, yeast, nematode, or fruit fly models; for longer-lived organisms there are obvious experimental difficulties.

### Example 1: 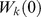 and 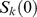 for Modifiers of Mutation and Migration Rate

#### Model 1: a simple two-locus model

We examine the behavior of 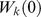 and 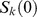 with a simple and well-studied model [16], [17], [18], [19], providing a straightforward illustration of several factors that influence survivability. In the model, the genotype is asexual and has two loci: 1) a major locus with possible alleles *A* and *a*; and 2) a mutator locus that controls the mutation rate at the major locus. The mutator locus has seven possible alleles *M_2_* through *M_8_*, where allele *M_m_* sets the symmetric mutation rate between alleles *A* and *a* at the major locus to 1×10^−*m*^. There are two possible environments, *E_A_* and *E_a_*. In environment *E_A_*, allele *A* is favored, and in environment *E_a_*, allele *a* is favored. A genotype with the favored allele has fitness 1+*s*, and a genotype with the disfavored allele has fitness 1. The selection coefficient *s* is set to 0.1 in all the experiments below. The population size, *N*, is fixed in any given experiment, but we also investigate its role in the evolutionary dynamics.

#### A baseline for comparison: infinite population size, with no environmental switching

**Figure 1:**
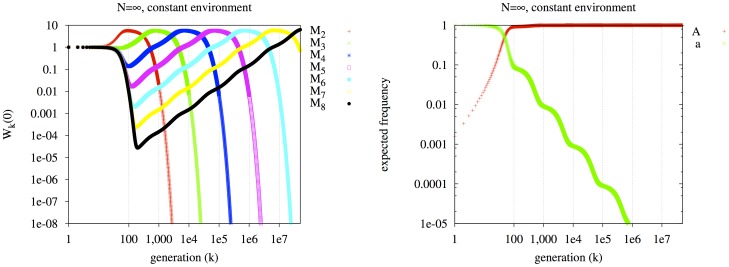
Infinite population size and constant environment. At left, 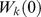, the *k*-fitness the seven mutator lineages (*M_2_* through *M_8_*). At right, the frequency of the two major alleles (*A* or *a*) at generation *k*.

Figure 1 plots the results of an experiment with infinite population size, and no environmental switching, which we use as a baseline for comparison with later cases. The environment is *E_A_* at the beginning of each experiment, and remains constant at *E_A_*. The population is initially equally divided into seven (out of the possible 14) genotypes: the seven mutator alleles are present at the mutator locus, but only allele *a* (the disfavored allele) occurs at the major locus.

At the left of Figure 1, we plot 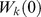, the *k*-fitness at *t* = 0 (the factor of increase *k* generations after *t* = 0) of the seven mutator lineages. All individuals are initially maladapted (since all possess allele *a* at the major locus, but the environment is *E_A_*). Lineage *M_2_* (which has the highest mutation rate, 1×10^−2^) initially converts its members to the favored *A* allele most rapidly; thus, by generation *k* = 100, lineage *M_2_* (red) dominates. However, by approximately this time, most of the members of lineage *M_3_* (green) also possess *A*, and are mutating back to *a* at a slower rate than lineage *M_2_*. Thus, both lineages are well adapted, but *M_3_* now has a smaller load of deleterious mutations, and overtakes *M_2_*, peaking by generation *k* = 1,000. This pattern (i.e., lineage *M_m_* superseding the current dominant lineage *M_m-1_*, and peaking around generation 10*^m^*) repeats through the end of the experiment at generation *k* = 5×10^7^.

At right, the frequency of the two major alleles (*A* or *a*) at generation *k* is shown. However, because the major allele is mutable, this frequency is not the frequency of descendents of individuals that *initially* possessed allele *A* or *a*. (Indeed, no individuals initially possessed allele *A*.) At the right of the figure, we see that the *a* alleles (green) gradually decrease in frequency, as 1) they mutate to *A* (red) – more rapidly for higher mutation rate lineages, and 2) the *A* alleles mutate ever more slowly back to *a* – as the lower mutation rate lineages eventually come to dominate.

#### The effect of population size

**Figure 2:**
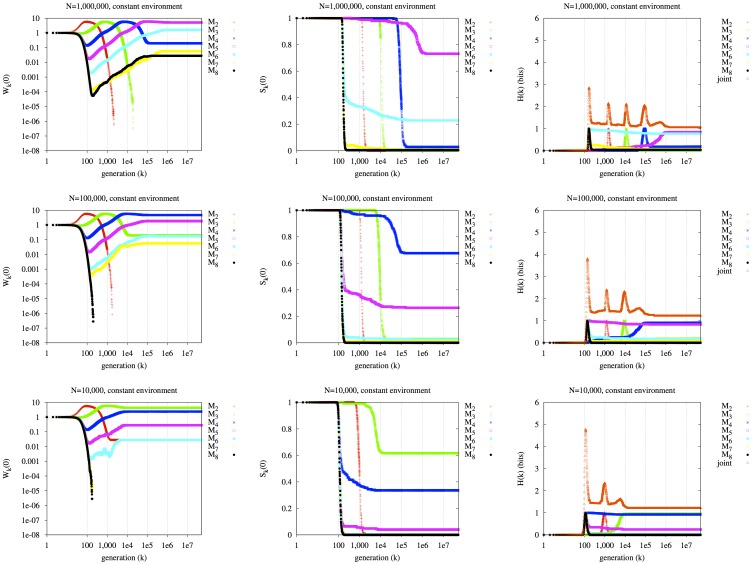
The effect of finite population size on the evolutionary dynamics of the seven mutator lineages. In rows: three values of *N* (*N* = 1,000,000, 100,000, 10,000). Left column: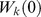. Middle column: 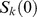. Right column: 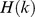. No environmental switching.

With finite population size, the short-term fitness becomes more important to long-term survivability. Each row of Figure 2 averages 250 experimental replicates. The environment is constant at *E_A_* and all individuals are initially maladapted (possessing allele *a* at the major locus). Three values of the population size, *N* = 1,000,000, *N* = 100,000, *N* = 10,000, are arranged in the three rows. In the left column, we plot the *k*-fitness at generation zero,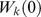. In the middle column, we plot the k-survivability at generation zero, 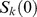. The right column plots our metric of predictability, 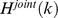, the joint survival entropy of all lineages, as well as the individual survival entropies, 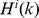.

The left panel of the top row of Figure 2 (N = 1,000,000) is similar to the infinite population case (Figure 1, left) until *k* approaches generation 5×10^5^. At this time, lineage *M_6_* (cyan) superseded *M_5_* (magenta) in the infinite population case, but it fails to do so here. The reason is that, early in the experiment when lineage *M_2_* (red) sweeps to dominance, *M_6_* approaches a very low expected count of members (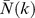, not shown). In some replicates, lineage *M_6_* drifts to extinction, which decreases its 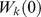 in the long term (at high *k*), so much that its 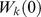 is lower than that of *M_5_* in the long term. In the center panel of the top row, the survival probability, 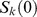, of lineage *M_6_* (cyan) drops drastically around generation 200, to about 40%, concurrent with the initial increase of *M_2_* (red). This sweep produces in *M_6_* about a 60% chance of going extinct early.

Extinction due to early maladaptation is even more apparent in the second row (*N* = 100,000) of the figure. In the left panel, *M_8_* (black) and *M_7_* (yellow) would be expected to dominate by generation 10^8^, and 10^7^, respectively, in an infinite population. Instead, both *M_8_* and *M_7_* go extinct in nearly all replicates by around generation 200. In the center panel of the second row, we see not only that *M_8_* and *M_7_* nearly always go extinct by generation 200, but also that *M_6_* (cyan) survives less than 5% of the time, and even *M_5_* (magenta) survives past generation 200 only about 40% of the time. In the center panel of the middle row, *M_2_* (red) and *M_3_* (green) go extinct around generation 2,000, and 20,000, respectively; this is due to high mutational load (relative to contemporary surviving lineages). The net result is that *M_4_* (blue) dominates in both expected fitness and survival probability before generation 1×10^5^.

In the third row of the figure (*N* = 10,000), a qualitatively similar dynamic obtains, with the net result that *M_3_* (green) is the lineage that 1) adapts rapidly enough in the short term to avoid short-term extinction, but 2) has a low enough mutation rate that its mutational load is less than all other surviving lineages (i.e., *M_2_* (red)). Thus, at low population sizes, short-term fitness (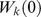 for low *k*) is important in determining the long-term dominant lineages, since lineages can be driven irreversibly extinct in the short term.

Note that in this example, all lineages had the same *immediate* (*k* = 1) fitness at *t* = 0 (i.e., 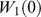): they were all initialized with the disfavored *a* allele. However, even if lineage *M_2_* had an initial fitness advantage by starting with, for example, 10% *A* and 90% *a* at the major locus, this initial “head start” would not cause it to dominate in the long term in all cases; it would still be “outrun” (in most cases) by other lineages due to its relatively high mutational load (not shown). Furthermore, in this example, all mutator lineages began with equal numbers of members; however, an initial population size advantage can also serve as a buffer against an initial decline due to poor short-term fitness (not shown).

The right column of Figure 2 contains plots of survival entropy. The entropy of the joint survival state, 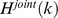, is plotted with orange triangles. In the long term, 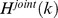 attains a value of just over 1 bit in the first row; note in the middle column (which plots 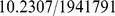), there are two lineages with a high chance of surviving in the long term: *M_5_* about 85% of the time, and *M_6_* about 15% of the time. One additional lineage, *M_4_*, survives a few percent of the time. This yields a “surprisal” in the joint outcome of about 1 bit in the long term; we are about as surprised as we are to see the outcome of a fair coin flip. Considering the variation of 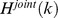 over time, we see several transient increases in uncertainty; the plots of 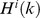 for the 7 lineages (other colors besides orange) make it clear that these increases are associated with the extinctions of individual lineages, which are temporally blurred across replicates. The right-hand plots in the second and third rows are qualitatively similar: 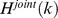 shows transient increases as one or more lineages go extinct, and a surprisal of about 1 bit in the long term. In the bottom right panel, the first jump (between generations 100 and 200) in surprisal is particularly large: this is because multiple lineages are simultaneously tending to extinction at this time, with uncertain ordering of the extinction events.

### The Effect of Environmental Switching at Mean Period T

**Figure 3:**
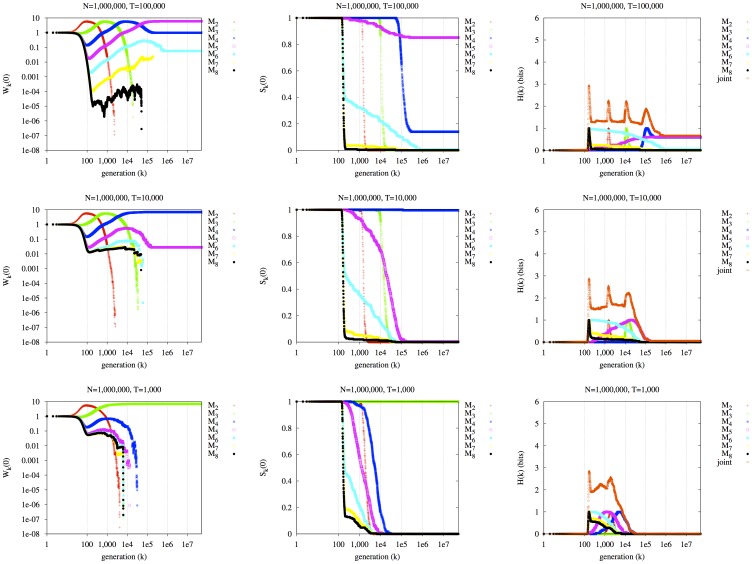
Effect of environmental switching on evolutionary dynamics of the seven mutator lineages. In rows: exponentially distributed environmental switching at three values of mean period *T* (*T* = 100,000, 10,000, 1,000). Left column: 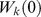. Middle column: 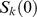. Right column: 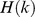. *N* = 1,000,000.

We next consider the effect of stochastic switching of environment at mean period *T*. In the following experiments, the length of each environmental “epoch” is τ generations, where τ is an exponentially distributed random variable with mean *T*. The environment switches from *E_A_* to *E_a_*, or vice versa, at the end of each epoch. In Figure 3, we plot 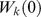 (left column), 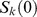 (middle column) and the entropies (right column) for three values of *T*: 100,000, 10,000, and 1,000. Previous work indicates that a lineage with mutation rate of approximately 1/*T* will eventually dominate [16], [17] if selection is strong enough [18] and “symmetric” [19], [20], or close to equal in the two environments, as it is here. The dynamic can be understood intuitively by considering that lineage *M_m_*, which produces mutation rate 10^−*m*^, strikes the correct balance of mutation rate between the early and late phases of each epoch of mean duration *T* = 10*^m^*, as follows: 1) early in each epoch, when most individuals in a lineage are maladapted at the major locus (because an environmental switch has just occurred), a high mutation rate will rapidly generate the alternate, adapted allele; however, 2) late in each epoch, when most individuals in a lineage are already adapted, a low mutation rate will generate fewer copies of the now-deleterious alternate allele at the major locus. The lineage with a correctly balanced mutation rate (approximately 1/*T*) repeatedly gains on its competitors during each epoch. Lineages with a low mutation rate (i.e., 10^−*m*^ <1/*T*) do not switch to the favored allele rapidly enough early on; and lineages with a high mutation rate (i.e., 10^−*m*^ >1/*T*) suffer late in the epoch from a high load of deleterious mutations.

Any particular replicate run will be qualitatively similar to the case of no environmental switches, until the first switch occurs, which is expected at generation *T* = 10*^m^*. Lineage *M_m_* is expected to see a net gain on its competitors up to this point. At this moment, a new epoch begins, again with expected length *T = *10*^m^*, which will again cause an expected gain for lineage *M_m_*. These repeated epochs of expected length *T* repeatedly favor lineage *M_m_* (in expectation), until all other lineages are driven extinct. Thus, in the three rows, lineages *M_5_* (magenta), *M_4_* (blue), and *M_3_* (green), respectively, dominate in the long term, for *T* = 100,000, 10,000, and 1,000. The population is large enough here (*N* = 1,000,000) that the long-term winner expected for the infinite population case is also the winner here, for each of the three rows. Note that in the top, center panel of Figure 3, lineage *M_m_* (*m* = 5 in this case) does not attain exactly 100% survival probability; this is due to the finite population size. For *N* = 10,000,000, it comes closer (not shown).

In the second and third rows of the right column of Figure 3, 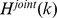 is very close to zero after a certain point. There is essentially no uncertainly in the population state in the long term in these runs: exactly one state is seen, with near-perfect repeatability. In the second row, this state is the fixation of lineage M_4_, and in the third row, it is the fixation of lineage M_3_. Note that in this model, a single lineage always fixes eventually (if the population is finite). However, in a more complex model that allowed multiple lineages to survive indefinitely, 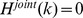 would still indicate perfect repeatability if, for example, the same two lineages always survived (comprising a single joint survival state).

#### Behavior of 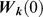 and 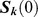 for a modifier of migration rate

**Figure 4:**
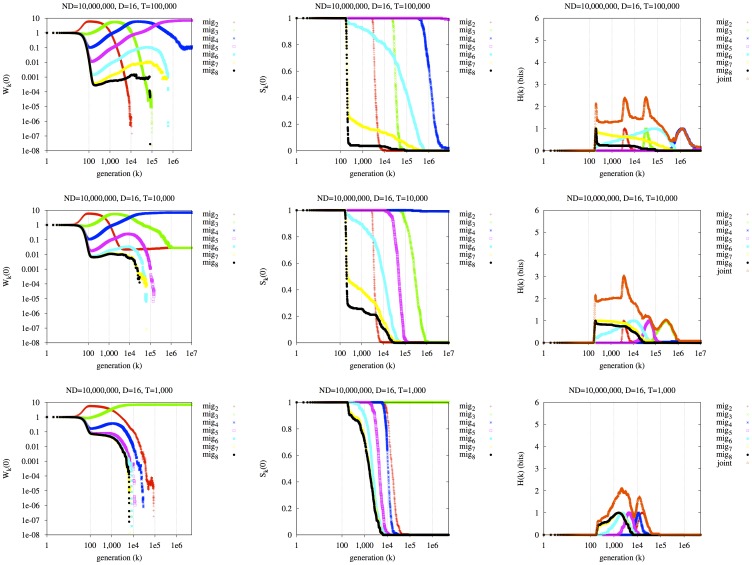
Effect of environmental switching on seven *migrator* lineages, *mig_2_* through *mig_8_*. In rows: three values of *T* (*T* = 100,000, 10,000, 1,000). Left column: 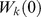. Middle column: 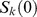. Right column: 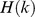. D = 16 demes, total population *ND* = 10,000,000. Mutation rate *µ* = 0.0.

In the next set of experiments, we construct a metapopulation of *D* demes. Each deme starts with a random environment, with equal probabilities of *E_A_* or *E_a_*. With probability 1/*T* per generation, each deme may switch to the other environment; the mean waiting period between switches is thus *T* generations. At any generation, we expect half of the demes to have environment *E_A_*, and half *E_a_*. Interestingly, just as a certain mean period of environmental switching can produce long-term selection for a certain *mutation* rate, it can also produce long-term selection for a certain *migration* rate [21], [22], in this spatially-structured setting. Here, the mutation rate is zero, and the *migration* rate is controlled by a modifier locus. Alleles *mig_m_*, where *m* ranges from two to eight, produce a migration rate of 10^−*m*^ per individual per generation in their carriers. Initially there are equal fractions of the seven lineages, *mig_2_* through *mig_8_*, in each deme, with all individuals possessing the maladapted allele for its local deme at the major locus. Individuals carrying allele *mig_m_* migrate from their current deme to any other deme with probability 10^−*m*^ per generation. In Figure 4, the migrator allele *mig_m_*, producing migration rate 10^−*m*^, where *T* = 10*^m^*, dominates in all three rows (*T* = 100,000, 10,000, 1,000) in the long term. The migration rate of 10^−*m*^ produces the appropriate balance between remaining in the current environment, and reaching a new one. The repeated environmental epochs at expected period *T* cause the lineage with the highest survivability over an epoch of length *T* to also have the highest long-term survivability, very repeatably.

### Example 2: 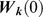 and 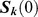 for a Modifier of the Genotype-phenotype Map

#### Modifiers of the genotype-phenotype map

In a previous paper [8], we distinguished between genes that vary rapidly (e.g., those that have a high mutation rate), and those that vary slowly (or not at all). The latter can be considered part of the genotype-phenotype (GP) map, which provides a long-lived context for the fast varying genes, fixing their phenotypic “meaning”, which is produced by epistatic interaction among all genes (slow and fast). Variation in the genes defining the GP map also alters the distribution of phenotypic variation, but because these genes vary only slowly, they provide an averaged evolutionary response over the long term [8]. We provide an example of a modifier of the GP map in a second computational model.

#### Model 2: two genetic vectors contributing to two phenotypic components

In the two-dimensional “angles and magnitudes” model [7], [8], the phenotype is a two-dimensional point on the plane. Two genetic factors – two two-dimensional vectors on the plane specified by the angle-magnitude pairs (*θ1*, *m1*) and (*θ2*, *m2*) – are summed to produce the phenotype. Thus each genetic factor can be considered to have a pleiotropic effect on two distinct phenotypic traits. We define α as the smaller of the two angles described by lines parallel to the two genetic vectors. Thus, α ranges from 0 to 90 degrees; an α of 90 indicates no overlap of gene action, and an α of 0 indicates complete overlap. A target point defines the optimal phenotype in a given environment and the target points are chosen from the uniform distribution over the unit disc. In the simulations described here, we select a different target point for each of 16 demes, and hold the targets fixed for the duration of a simulation run. Fitness (*w*) increases as the distance (*d*) decreases between the phenotype and the target point in its deme according to *w* = 1/(*ss*+*d*), where *ss* modulates selection, and is here fixed at *ss* = 0.01.

Each deme initially contains only cloned copies of a random low-α (α = 5) individual, one distinct clone per deme. The population size is *N* = 64 per deme, and there are *D* = 16 demes, for a total *ND* = 1024 individuals. With no migration among demes, the clones pre-adapt to their local targets for a certain number of generations. The angles of the genetic vectors are not permitted to mutate, *µ_a_* = 0.0; the magnitudes mutate at rate *µ_m_* = 0.04. After the pre-adaptation phase, 50% of the existing low-α individuals in each deme are replaced by clones of a random high-α (α = 85) individual, one distinct clone per deme. Thus, the low-α individuals are initially better adapted, but the high-α individuals produce a superior distribution of variation. Migration is initiated at a rate of 0.01 per individual per generation, and evolution proceeds for 100,000 generations.

**Figure 5:**
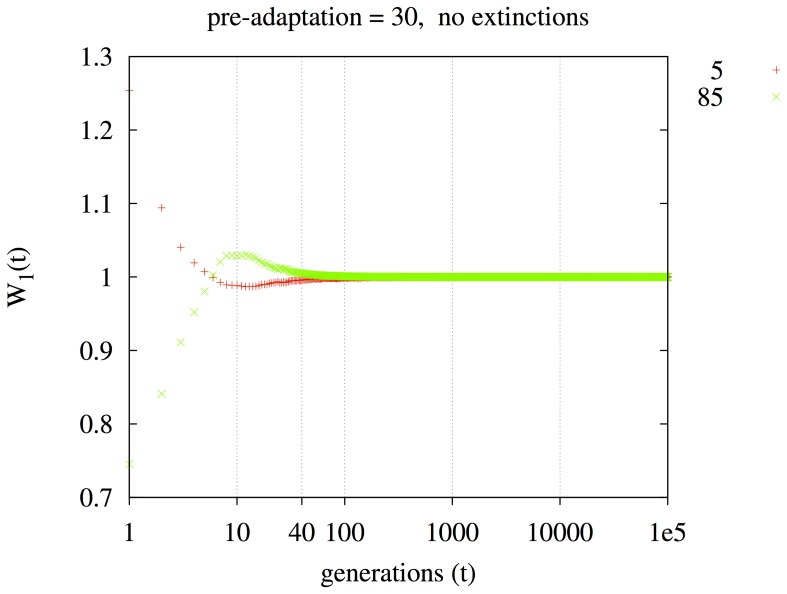
An initially better-adapted lineage (red, α = 5) has a higher 1-fitness, 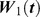, for *t*≤5. However, a lineage that produces more high-fitness mutants (green, α = 85) attains a higher 1-fitness, 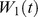, for 5<*t*<200.

In Figure 5, the initial α = 5 individuals pre-adapt for 30 generations in their sixteen demes. 50% of them are replaced by clones of sixteen individuals with α = 85, one distinct clone per deme. Migration begins and the system evolves for 100,000 generations. The 1-fitness, 
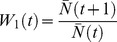
, for the pre-adapted α = 5 strain (red) is higher for *t*≤5. However, the α = 85 strain (green) produces more high-fitness mutants and its 1-fitness, 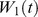, becomes higher for 5<*t*<200. Note that in Figure 5, the X-axis indicates a range of *t* (while *k* is fixed at 1), unlike other figures.

**Figure 6:**
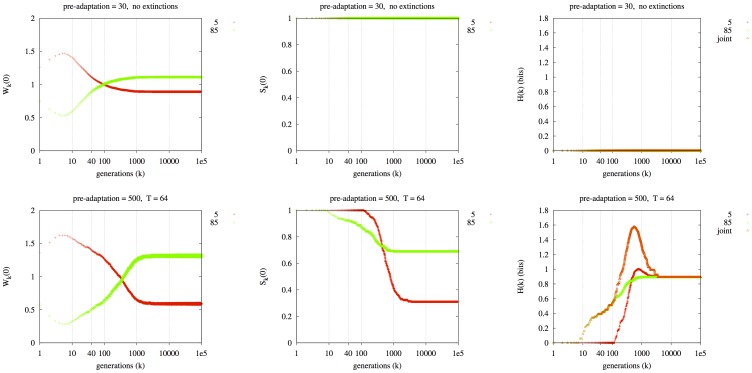
An initially better-adapted lineage (red, α = 5) competes against a more lineage that produces better variation and therefore adapts quicker (green, α = 85). First row: short pre-adaption (30 generations), but no per-deme extinctions. Second row: long pre-adaptation (500 generations), per-deme extinctions at mean period T = 64. D = 16, ND = 1024.

In contrast, in Figure 6, the X-axis indicates a range of *k* (while *t* is fixed at 0). In Figure 6, the left column plots 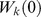, the *k*-fitness for *t* = 0, and the middle column 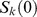, the *k*-survivability for *t* = 0. The right column plots 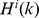 for each lineage and 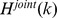. The two rows of Figure 6 contrast two situations. In the first row, the initial α = 5 individuals pre-adapt for 30 generations. Then 50% of each deme is replaced by clones of sixteen individuals with α = 85, one distinct clone per deme. Migration occurs and the system evolves for 100,000 generations. The title “no extinctions” over each panel in the top row indicates that there are no periodic extinction events that wipe out all individuals in a deme. At left, we see that 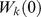 for the α = 5 lineage (red) initially increases; this is due to the high initial 1-fitness (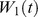 for low *t*) of the α = 5 lineage (which was pre-adapted to its local target in each deme); however, the α = 85 (green) lineage adapts more rapidly. By generation 100,000, the α = 85 strain has attained a slightly higher 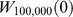
value than the α = 5 strain, on average over 300 replicates. The benefit of the superior distribution of variation produced by the α = 85 lineage was approximately matched by the benefit of 30 generations of pre-adaptation of the α = 5 lineage, such that their average long-term fitnesses, 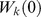 for high *k*, are roughly equal. Interestingly, as shown by 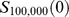 in the top right panel, both lineages avoid long-term extinction in the metapopulation in all replicates: in each replicate, each lineage has fixed in some of the *D* = 16 demes (not indicated in figure), thereby avoiding extinction in the metapopulation; this joint survival state (survival of both lineages) is very repeatable (zero joint entropy).

In the second row, there is a longer pre-adaption period for the α = 5 lineages: 500 generations, but each deme also experiences an extinction after an exponentially distributed waiting time with mean *T* = 64 generations, which eliminates all adapted incumbent individuals; new migrants must adapt to the target of their new deme. This decreases the pre-adaptation advantage of the α = 5 lineage over the long term; the production of new variation after *t* = 0 becomes more important because the individuals that survive over the long term are those that are able to repeatedly migrate and adapt to a new target. Thus, despite the longer pre-adaptation period of 500 generations, the two lineages end up again with (fairly) similar long-term fitness, 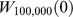. Indeed, the two plots of 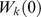 in the two rows are qualitatively similar; the two curves cross each other in *k*-fitness in both cases. However, the two plots of 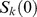 are quite dissimilar. At bottom right, because the α = 5 lineages are initially very well adapted, the α = 85 (green) lineages go extinct in the entire metapopulation early on in many replicates; for example by generation 1000, the α = 85 lineages survive in only about 70% of replicates. However, the repeated extinctions force all individuals to migrate and adapt, or die. The α = 85 lineages tend to adapt faster, by producing a superior spectrum of variation (see [8]). Thus between generation 1,000 and 10,000, the α = 5 (red) lineages go extinct in the entire metapopulation in many replicates; they survive in only about 35% of replicates in the long term. There are about 0.9 bits of uncertainty in the long term: either the α = 5 lineage eliminates the α = 85 lineage in the short term; or it fails to do so, and is subsequently outcompeted by the faster-adapting α = 85 lineage. Thus, although the two sets of conditions (two rows) appear superficially similar in terms of 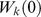, their different dynamics are revealed by the plots of 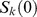 and 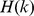.

## Discussion

Other authors have considered the survivability of lineages. Whereas Thoday [23] suggests that one should pick an arbitrary, long time duration, “such as 10^8^ years”, Slobodkin [24] insists that the, “short-term probability of survival is in fact the only legitimate measure of evolutionary health.” Cooper [25] proposes that one should consider the Expected Time to Extinction (ETE). Each of these proposals throws away information. There is no single *k* that is natural to prefer in general, and retaining 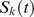 for all *k* ≥0 illuminates the evolutionary dynamic, as shown by our examples above.

### Barriers to the Increase of Long-term Survivability, and How they are Sometimes Overcome

There are two major barriers to the evolutionary increase of the long-term survivability of a lineage. First, sub-lineages (and also individuals, in sexual populations) may compete. Second, selection in the short-term may act against the long-term survival of the lineage.

#### Competition between sub-lineages

Sub-lineages may compete to the extent that one drives the other extinct. This may be to the detriment of the lineage, which would, in general, benefit from numerically large and genetically diverse sub-lineages. A more favorable outcome for the lineage would be for one of the sub-lineages to migrate elsewhere, or for either to diversify so that they would not be in direct competition. On the other hand, the churning of short-term evolution will sometimes produce variation that improves survivability in the long term for “exaptive” reasons. This is analogous to a “random” mutation producing variation at the organismal level. As Gould and Lloyd [11] state, “The species-individual, by not suppressing selection at lower levels within itself, thereby maintains a large reservoir of… traits that are built at these lower levels but provide potential exaptive benefit to the species.”

#### Conflict between selection at different timescales

If a lineage possesses variation that is short-term-adaptive, but is long-term-maladaptive, sub-lineages may respond to short-term selection, but in such a way as to produce long-term decline in the lineage. For example, consider a lineage that may reproduce either sexually or asexually. A sub-lineage might begin to reproduce exclusively asexually, which benefits it in the short-term; however, in the long run, asexual lineages are more likely to go extinct in a changing environment than sexual lineages [26]. The asexual sub-lineage may even exclude other sexual sub-lineages, and then subsequently go extinct, causing the extinction of the entire parent lineage.

A higher-level entity (e.g., a lineage) can influence the short-term evolution of a lower level entity (e.g., a sub-lineage) by *depriving the lower-level entity of short-term-adaptive variation that is long-term-maladaptive*. Some (parent) lineages, by chance, will evolve genetic barriers that prevent easy switching to asexual reproduction; this effectively “enslaves” [27] their members to reproduce only sexually. (For example, a mutation might disable one of the molecular systems needed for asexual reproduction, and compensatory mutations could be slow to repair a complex mechanism.) This short-term “barrier” could equally well be considered a long-term “adaptation”. Over the long term, selection among lineages eventually removes those that failed to evolve genetic systems that properly balance long- and short-term adaptation.

### A Relevant *in vivo* Experiment

Woods et al. [28] isolated strains of bacteria from a long-term bacterial evolution experiment [29] in which one strain, named EW (for “eventual winner”), was at one point less fit, but later more fit, than another strain, named EL (or “eventual loser”). When they “replayed” evolution in multiple replicates, they found this pattern was consistent. They isolated random clones of each strain, propagated them independently for 883 generations, and measured the short-term fitness (over one day, or approximately seven generations) of the two strains at two time points, *t* = 0 and *t* = 883. Using our terminology, they found at *t* = 0 that EL was 6.3% fitter in terms of relative 7-fitness (*k* = 7 generations). At *t* = 883, EW was 2.1% fitter in terms of relative 7-fitness. Thus, they measured 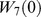 and 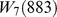, establishing that EW initially had a lower short-term fitness, and later had a higher short-term fitness, repeatably. However, the long-term (e.g., *k* = 883) fitnesses are not measurable from the experiments reported. The long-term fitness could be measured in principle, e.g., by genetically tagging two lineages, and then counting the frequency of tags at some *k*≫1, in replicate experiments.

### Survivability is Fundamental

Lineages do not always succeed because they acquire new adaptations; sometimes it is advantageous to remain the same. Sometimes it is more important to physically disperse, or to diversify genetically. Survivability can describe and predict long-term outcomes. The lineages that we observe today can be described as *those that have survived*. Some of these survivors were lucky, surviving due to a confluence of improbable events in a particular realized instance of natural history. However, if it were possible for us to repeatedly “replay the tape” [30] of natural history, with slight random variations each time, we think it likely that certain lineages would repeatedly survive. In this sense, it is not a tautology to say that *those that have survived will tend to be those that had high survivability*, if we could measure this in many similar replicates of natural history.

It is impossible in practice to measure the survivability of now-extinct organisms. We cannot “replay the tape” on such a grand scale. The standard 1-generation fitness is also difficult or impossible to measure in many cases, yet it is clearly an indispensible theoretical, and sometimes practical, tool for quantitative understanding of evolution in the short term. The *k*-fitness and *k*-survivability are also theoretical, and sometimes practical, tools, but they are applicable to any time span. Just as we look for the signature of selection (as measured by the 1-generation fitness) in data from contemporary and extinct organisms, so might we also expect to see the signature of long-term selection on lineages.

In nature, multiple “replicates” of similar evolutionary contests may occur in temporally repeated fashion, during cyclical fluctuation of environments. Such replicates may also occur in parallel across spatially structured environments, when, for example, a lineage invades a new landscape with patchy spatial variation. Whether or not it survives will depend on whether it can repeatedly win similar evolutionary contests in the distinct patches. There will be a component of chance, especially right after invasion when absolute numbers are small; but there may also be significant repeatability, producing a meaningful long-term fitness. Such spatially structured environments may provide opportunities to observe multiple evolutionary replicates *in vivo*.

The repeatability of evolutionary experiments will depend on the distributions I (the distribution of initial states) and E (the distribution of environments), and also upon the constraints of the genetic system. In a simple abstract model of one locus with two possible alleles, the evolutionary possibilities are limited. More complex *in silico* models, and biological models, may permit a multitude of evolutionary pathways; however, the variation that a lineage can generate is not unlimited. Certain “basins of attraction” may lead to parallel evolution across replicates. For example, selection on continuous traits like body size may easily produce repeatability. In contrast, the genetic constraints of a lineage might be such that it must fix a particular mutation before it can go on to make further adaptations. If this first mutation fixes in only 10% of replicates, such subsequent adaptations can happen at most 10% of the time. Thus it is an interaction of I, E, and the genetic constraints of the lineage that determine repeatability.

We do not see the concept of evolvability as generally quantifiable. No phenotype is fit in *every* environment. Clearly, some context or distribution of environments must be specified. Thus, just as the standard fitness is specific to a particular environment, the *k*-fitness and *k*-survivability are specific to a particular distribution of environments (E).

Our examples have involved several modifiers of the distribution of variation: a modifier of mutation, a modifier of migration, and a modifier of the GP map. However, other more complex phenomena may affect survivability, including 1) rates and mechanisms of mutation, recombination, and other genetic operators, 2) dispersal mechanisms, 3) the genotype-phenotype map, 4) sexual reproduction, 5) epistatic inheritance, and 6) niche construction [31], as they evolve in temporally and spatially varying environments. Although the evolution of these disparate phenomena involves well-understood microevolutionary rules, they are also subject to the macroevolutionary constraint of long-term survivability.

## Methods

For both model 1 (the two-locus model) and model 2 (the angles and magnitudes model), generations do not overlap, and the order of operations is: change of environment (if any); extinctions (if any); computation of fitness; reproduction; mutation; migration (if any). The expected number of offspring (fitness) is proportional to the “raw fitness” as specified for the respective model, with the mean fitness normalized to one.
